# Salutatory

**Published:** 1866-01

**Authors:** 


### SALUTATORY.

In assuming the relation in which we now come before the readers of the JOURNAL, we involuntarily glance over the twenty-two volumes, which remain as evidence of the fidelity and successful efforts of those who have preceded us.

And as we follow through the names of those who have been so intimately identified for so many years with the interests of our profession in the Northwest, who have written and taught so faithfully, that their influence is felt and honored by thousands of our co-laborers in the practice of medicine, we cannot but feel some misgiving in attempting to carry forward the work which has now been transmitted to us.

We can only assure our friends that we will devote our best efforts to maintain the JOURNAL in the position it has so long occupied; but must earnestly ask such aid as the profession can give us.

There are probably not four complete files of the JOURNAL now in existence. Comparatively few physicians in this city or in the country are acquainted with its history. A few facts connected with its origin and growth, therefore, may not be uninteresting to many of our readers.

The first number was published in April, 1844, under the title of "The Illinois Medical and Surgical Journal," J. V. Z. Blaney, M. D., Professor of Chemistry in Rush Medical College. being editor. The JOURNAL was continued two years as a monthly, containing but sixteen pages.

In April, 1846, under the direction of Professors Blaney, Brainard, Herrick and Evans, the title was changed to "The Illinois and Indiana Medical and Surgical Journal." It contained ninety-six pages, and was issued for two years simultaneously, once in two months, at Chicago and Indianapolis.

In 1848 a "new series" was commenced as "The North-Western Medical and Surgical Journal," published in Chicago, and was continued as a bi-monthly of ninety-six pages for four years—under the charge, first of Professors Herrick and Evans, then of Prof. Herrick and Dr. E. G. Meek, and finally of Prof. Evans alone.

For the following five years the JOURNAL was published monthly, first under the direction of Professors Herrick and Johnson, subsequently of Professors Davis and Johnson, and finally of Prof. Davis.

At the end of volume fourteen, (1857,) we find the following statement in the editorial: "The present title was chosen, when Chicago was really close to the Northwestern border of our magnificent Union; but at present it would be much more applicable to a journal published in Oregon or Washington territory. Hence, the January number will be issued under the title of "The Chicago Medical Journal."

Since 1858 the JOURNAL, under this title, has been edited, for different periods, by Drs. Davis, Byford, Brainard, Dyas, Powell, Miller and Ingals.

The editorials of these twenty-two volumes present no insignificant history of the development of our profession, not only in the Northwest, but throughout our whole country. They convince us that our medical journals are the medium through which most members of the profession usually become acquainted with the existence and reputed value of new publications in the different branches of medical science; that they record the most important facts connected with our social and educational interests, and with the growth of our colleges, hospitals and associations.

We are consequently not among those who think lightly of the good which is accomplished by the medical journals of this country. Like the results of all human labor, they are undoubtedly imperfect, and yet it would be difficult to imagine the position of the medical profession if they should all cease to exist.

It will be our aim, therefore, to secure for our readers original papers on important medical subjects; to present valuable selections from foreign journals, and from our large list of American medical periodicals; to obtain reports of interesting cases, observed in the hospitals, infirmaries and dispensaries of Chicago, and to call the attention of the profession to new and valuable works in the various departments of medical science.

While it will be our chief object to aid in promoting the best interests of the profession in the culture of true science, we shall also endeavor to publish such intelligence, not strictly scientific, as may be of particular interest to the practising physicians of the Northwest.

We shall be under obligations to those friends, either in the city or country, who will favor us with such original articles as may aid us in rendering the JOURNAL acceptable to our readers.

